# Comparison of Carrying Angle of Elbow between Patients with and without Lateral Epicondylitis: An Observational Study

**DOI:** 10.31729/jnma.8863

**Published:** 2025-01-31

**Authors:** Sarik Kumar Shrestha, Manoj Kandel, Bhanubhakta Chalise, Keshav Mani Gautam, Sunil Panta, Pritam Chaudhary, Suman Subedi, Prakash Darjee

**Affiliations:** 1 Department of Orthopedics and Trauma Surgery, Bharatpur Hospital, Bharatpur, Chitwan, Nepal; 2 Department of Orthopedics and Trauma Surgery, Gorkha Hospital, Gorkha, Nepal

**Keywords:** *carrying angle*, *lateral epicondylitis*, *tennis elbow*

## Abstract

**Introduction:**

Lateral epicondylitis is a common cause of elbow pain, characterized by pain on the lateral side of the elbow joint. Its multifactorial etiology may result from repetitive micro-trauma, overuse of the forearm extensor muscles, use of vibrating tools, and anatomical variables causing lateral wear. In case of increased carrying angle of elbow, the course of extensor carpi radialis brevis during movement can be altered, thus increasing the tension on the extensor carpi radialis brevis tendon and increasing the risk for lateral epicondylitis. This study aims to find out the difference in carrying angle of elbow in patients with and without lateral epicondylitis.

**Methods:**

An observational cross-section study comparing carrying angle of elbow in patient with and without lateral epicondylitis was performed after ethical approval from the Institutional Review Committee (Reference number: 080/081-019) Patients presenting who had undergone radiographic examination of the elbow were recruited in the study and divided into the lateral epicondylitis group and the normal group. The mean carrying angle of elbow in both groups was compared using the parametric test.

**Results:**

This study included 82 participants, with 41 participants in the lateral epicondylitis group and 41 participants in the normal group. The mean carrying angle of elbow among patients with lateral epicondylitis group was 14.35±1.4 degrees; and the normal group, it was 12.40±1.26 degrees (p<0.001).

**Conclusions:**

The carrying angle of elbow in patients with lateral epicondylitis was significantly more than those without lateral epicondylitis.

## INTRODUCTION

Lateral epicondylitis (LE) is a common cause of elbow pain, characterized by pain on the lateral side of elbow joint.^1-3^ The etiology is multifactorial and may result from repetitive micro-trauma, overuse of forearm extensor muscles, use of vibrating tools, and anatomical variables leading to excessive varus stress on extensor carpi radialis brevis (ECRB) tendon and causing lateral wear.^4-7^ When the carrying angle of elbow (CAE) is increased, the course of ECRB during movement can be altered, thus increasing the tension on the ECRB tendon and increasing the risk for LE.^8^

Only a few studies have evaluated the carrying angle of the elbow as the etiologic factor in lateral epicondylitis.^3,8,9^ The conclusion of these studies also seems to be variable.^3,8,9^ Therefore, this study aims to find if the CAE is significantly different in patients with LE compared to those not having LE.

## METHODS

This was an observational cross-section study conducted at Bharatpur Hospital, which is a tertiary-level hospital located in Bharatpur, Nepal. The study was conducted from July to November 2024 after ethical approval from the Institutional Review Committee (Reference number: 080/081-019). The sample size was calculated using Epi Info, version 7 using the mean and standard deviation of the carrying angle of elbow in the LE group and normal group in the previous study.^3^

The sample size for two independent samples with the continuous outcome (n) = 2 × [(Z 1- α/2+ Z 1-β)/ES]

Z 1- α/2 = 1.96 for 95% Confidence Interval

Z 1-β = 0.84 for Power 80%

Mean of Group 1 (μ1)= 13.80

Mean of Group 2 (μ2)= 15.90

Standard Deviation of Group 1 (SD1)=3.17

Standard Deviation of Group 2 (SD2)=3.60

Pooled standard deviation (σ) = √ (SD1^2^ + SD2^2^ / 2)

=3.39

Effect size (ES) = μ1 - μ2|/α = 0.62

Sample size (n) = 2 × [(Z 1- α/2+ Z 1-β)/ES]2 = 40.86 = 41

Sample size of LE group= 41

Sample size of Normal group= 41

Total sample size= 82

Convenience sampling method was used. The study population in the LE group included patients presenting to the Orthopaedics outpatient department (OPD) having a clinical diagnosis of LE and providing consent for the study. Similarly, the study population in the Normal group included patients not having LE, but having X ray of elbow done for other reasons and providing consent for the study. Patients less than 18 years and over 60 years, patients with a history of prior surgery or fracture on the same limb, and reduced range of motion of elbow were excluded from the study. The patients in normal group with previous history of LE were also excluded.

Diagnosis of LE was made in patients with tenderness over the lateral epicondyle of the elbow with positive results of the Cozen test.^3^ The normal group included patients who had undergone radiographic examination of the elbow for other reasons and had no history of lateral epicondylitis in the past. CAE was measured on an anteroposterior (AP) radiograph of the elbow taken with the forearm in supination and the elbow in extension. We drew a line connecting the two midpoints of the distal humerus- at the distal metaphysis and the distal third of diaphysis^10^. A line connecting the midpoints of the proximal ulna at two points- at the level of radial tuberosity and the ulna's most proximal ossification level was also drawn.^10^ Then, the angle between the two lines was measured. CAE was measured by two different orthopaedic surgeons and the mean of the measurements by two orthopaedic surgeons was taken as the CAE.

The inter-rater variability and comparison of age, sex, side involved, and dominant side was done for internal validity. The mean CAE was compared using T test and p value less than 0.05 was considered statistically significant. The collected data was entered in Microsoft Excel, which was used later for data analysis. Data analysis was done using Epi Info version 7 and SPSS version 30. The patient's personal information is kept confidential and data files are saved in a passwordprotected computer.

## RESULTS

This study included 82 participants with 41 participants in the LE group and 41 participants in the Normal group. The mean age of participants was 42.79±11.14 years in total. There were 42 (51.22%) males in total, 50 (60.98%) with right elbow involvement, and 79 (96.34%) had dominant right side in total (Table 1).

A comparison of age, sex, side involved, and dominant side was done for internal validity. The age of patients in the two groups was significantly different while other variables were not found to be different in the two groups (Table 1).

The mean CAE among patients with LE was 14.35±1.40 degrees; in the normal group, it was 12.40±1.26 degrees. This difference in CAE in the two groups was found to be statistically significant (p <0.001) (Table 1).

**Table 1:** Comparison of participants in the LE group and Normal group

Characteristics		Total Participants N(%)	LE Group (n=41) N(%)	Normal Group (n=41) N(%)	P value
**Number of participants**		82 (100)	41(50)	41(50)	
Mean age^~^		42.79±11.14	46.24±9.59	39.34±11.62	0.004
Sex^*^	Male	42 (51.22)	20 (48.78)	22 (53.66)	0.659
	Female	40 (48.78)	21 (51.22)	19 (46.34)	
Side	Right involved^*^	50 (60.98)	28 (68.29)	22 (53.66)	0.174
Left		32 (39.02)	13 (31.71)	19 (46.34)	
Dominant side^~^	Right	79 (96.34)	40 (97.56)	39 (95.12)	0.556
	Left	3 (3.66)	1 (2.44)	2 (4.78)	
Mean CAE		13.38±2	14.35±1.40	12.40±1.26	<0.001

~ Chi-Square test

* T Test, LE=Lateral Epicondylitis

A comparison of CAE measured by two different observers was done to test for inter-observer reliability. The interclass correlation was found to be 0.94. (Table 2).

**Table 2:** Inter-observer reliability in the measurement of CAE

		Mean	Interclass correlation
CAE^~^	Observer 1	13.34±1.72	0.94
Observer 2	13.41±1.63	

~ CAE = Carrying angle of elbow

## DISCUSSION

A statistically significant difference was seen in the CAE between those with LE and in the normal group in our study. There have been many studies that have assessed the relationship between CAE and upper extremity injuries and diseases.^11,12,13,14^ A study done on professional baseball players did not find the CAE to be associated with an increased risk of injury.^11^ Similarly, another study done on female recreational tennis players did not find the CAE to be different in symptomatic participants with LE and the control group.^12^ However, another study found CAE to be an independent risk factor for non-traumatic ulnar neuropathy.^13^ Another study also suggested that CAE can be used in the diagnosis of epicondylitis.^14^

The number of studies done to test the relationship between CAE and LE in the absence of other etiologic factors is however limited.^3,8,9^ The conclusion of these studies also seems to be variable, with some studies showing an association between CAE and LE while other studies do not show such association.^3,8,9^ The results of our study show that the CAE is significantly higher in the LE group compared to the Normal group. In contrast to our study, the results of one study showed a significantly decreased CAE in patients with LE compared to the normal group, although the pathophysiology could not be described.^3^ However, another study stated that increased CAE is associated with increased incidence of LE and they stated that increased CAE may contribute to the development of LE by increasing the tension on the ECRB tendon.^8^ The results of this study align with the findings of our study. Another study also showed significantly higher CAE in the LE group than the normal group and concluded that increased CAE in patients with LE is due to adaptive changes resulting from compressive forces on the lateral aspect of the elbow.^9^ The findings of this study was also similar to what we found in our study. Despite multiple factors involved in the development of LE, we have found that increased CAE can contribute to the development of LE.

In this study, both groups were comparable as there was no statistically significant difference between gender, side involvement, and dominant side in both groups. However, there was a statistically significant difference in age (46.24±9.59 years in the LE group vs 39.34±11.62 years in the normal group). However, we did not consider this difference in age to be a significant confounder since we have only included patients between the ages of 18 to 60 years in our study and CAE stabilizes after the age of 16 years.^15^ Other studies have also shown that the CAE gradually increases in children and reaches maximum value at puberty, then it remains stable throughout adulthood.^16-18^ Therefore, the difference in age in the two groups can be considered not to be significant as we have only included adults between the ages of 18 and 60 years in this study.

Also, the high inter-rater reliability in this study validates the measurement of CAE. Other studies have also shown high inter-observer reliability in the measurement of CAE.^19,20^

Most studies have shown that LE results due to overloading of the origin of the ECRB tendon.^4,5^ Its etiology is multifactorial and may be caused by repetitive micro-traumas, overuse of the forearm extensor muscles, and use of vibrating tools.^1,6^ Anatomical variables that cause lateral wear may also contribute to the development of LE.^3,7,11^ However, the mechanism by which the anatomical variables result in the development of lateral epicondylitis is still unclear.

The CAE can be defined clinically as the angle between the arm and forearm with the elbow extended and the forearm supinated.^8^ Radiologically, it is defined as the angle formed between the longitudinal axis of the distal humerus and proximal ulna on the AP view of the elbow taken with the elbow extended and forearm supinated.^8^ The normal range of CAE is 5°-15°, which is slightly more in females than in males.^8^ Functional impairment such as reduced range of motion of elbow joint, ulnar neuropathy, and early development of arthritis of elbow joint may occur from abnormalities in CAE.^8^

The exact pathophysiology behind how increased CAE leads to the development of LE is still unclear. However, many authors have given their views on the mechanism of causation of LE due to increased CAE. Changes in the CAE can change the tension on the ECRB tendon and thus change the abrasive and pressurizing forces over the ECRB tendon. As said earlier, increased CAE would alter the course of the ECRB tendon during movement of the forearm and elbow from supination-flexion to pronation-extension position. This would result in increased tension on the ECRB tendon, hence increasing the risk of lateral epicondylitis.^8^

Some studies have also shown the influence of other anatomical factors in the development of LE. The LE is associated with increased pronation of the forearm and reduced flexion of the wrist in symptomatic tennis players.^8^ Other anatomical variables that have been found important in the development of LE are anterior translation of the radial head and lateralization of the extensor carpi radialis longus (ECRL) tendon. These anatomical variables also increase the pressure on the ECRB tendon which can result in increased strain on the extensor muscles of the forearm.^7,8^ The result here also seems to be a greater strain on the ECRB tendon. From the findings from various studies and the above discussion, it is clear that anatomical factors and changes in the CAE are significant contributing factors in the etiology of LE.

This study does have a few limitations. Our study included patients from different occupational groups and since repetitive micro-trauma can result in LE, the inclusion of patients from various occupational groups can also be considered as a limitation. We also did not assess the relationship between different activity levels and CAE. Several studies have found that the incidence of LE is higher in people having physically demanding jobs.^21,22,23^ The activity level of the patients was not considered in this study. Further studies evaluating the effect of occupation on development of LE in Nepal can help better understand the role of occupation on the pathophysiology of LE. Also, we have found significant differences in the age of patients in the two groups, which we did not consider to be a significant confounder since studies have shown that the CAE stabilizes after the age of 16 years.^15^

Another limitation is that we have not performed advanced imaging such as computed tomography (CT) or magnetic resonance imaging (MRI) in patients with LE and only evaluated the CAE on plain radiographs. MRI can be helpful in the evaluation of patients with a elbow pain and can identify ECRB tendon tears.^24^ Also, the CAE can be more accurately measured using CT scans and also has high reliability.^25^ However, we have not used advanced imaging techniques such as MRI and CT scan in the evaluation or diagnosis of patients with LE. Future studies focusing on the role of such advanced imaging techniques for the evaluation of patients with LE can help us better understand the role of anatomical and physiological variables in LE.

## CONCLUSIONS

The carrying angle of elbow in patients with lateral epicondylitis was significantly more than those without lateral epicondylitis.
